# Is gambling like a virus?: A conceptual framework and proposals based on empirical data for the prevention of gambling addiction

**DOI:** 10.1186/s12889-023-16610-x

**Published:** 2023-09-01

**Authors:** Mariano Chóliz

**Affiliations:** Gambling and Technological Addictions Research Unit, Psychology School, University of Valencia, Avda. Blasco Ibáñez, 21, 46010 Valencia, Spain

**Keywords:** Gambling disorder prevention, Public health, Gambling policies, Gambling addiction, Responsible gambling

## Abstract

**Objective:**

The objective of this study is to present a conceptual framework for the prevention of gambling disorder and try to corroborate some of its postulates. The assumption of gambling as if it were acting like a virus may have important considerations in terms of preventing gambling disorder in society and, therefore, it could be a relevant public health issue.

**Background:**

Like COVID-19, gambling disorder is a disease which is caused by the action of an external agent. The external agent was already in existence, but certain environmental conditions (absence of regulatory measures based on the prevention of gambling disorder) favored its propagation. Regarding immunization, for SARS-CoV-2, it is obtained through vaccination and prevention of exposure. However, it is unlikely that immunization can be developed for any gambling addiction prevention program to immunize everyone who is exposed to the “gambling virus”. So, in the case of gambling disorder, preventive strategies should rather prevent gambling from affecting most people by limiting availability (supply) and accessibility (ease of access) to gambling.

**Study design:**

This research is a quasi-experimental investigation aimed to evaluate the effects of anti-COVID measures on the frequency of gambling and evolution of gambling disorder. The present study analyzed gambling patterns and the problems caused by gambling in 2,903 people, including those who were at-risk gamblers or had a gambling disorder.

**Results:**

In general terms, restrictive measures to combat COVID-19 worked to prevent the consolidation of gambling habits and the appearance of gambling disorder, but they did not seem to be sufficient for people who already had this disorder. The most affected games were electronic games machines (EGMs) that took place in public places (gambling halls, bars and restaurants, etc.).

**Conclusions:**

The findings of this work support the hypothesis that, just as the SAR-CoV-2 virus is responsible for the global pandemic of COVID-19, which can only be stopped with vaccines and social distancing, in the case of gambling, the absence of an effective vaccine for "gambling virus" can lead to an epidemic of gambling disorders in societies, if the environmental conditions that are favorable for the spread of such virus are not modified. Some preventive strategies that can be useful from a public health frame of reference are suggested.

## Introduction

The assumption of gambling as if it were acting like a virus may have important considerations in terms of preventing gambling disorder in society and, therefore, it is a relevant public health issue. So, the comparison between gambling and SARS-CoV-2 seems appropriate to guide health policies that aim to prevent gambling disorder, just as they have been taken worldwide for the prevention of COVID-19.

Gambling disorder shares some characteristics of infectious viral spread, such as that of the COVID-19 pandemic. The consideration of gambling as a virus is metaphorical, but it seems adequate for describing the increasing prevalence of gambling disorder in countries where gambling has been legalized and promoted; just as for COVID-19, preventive measures must be developed for this disorder.

First, gambling disorder shares some of the characteristics of infectious spread caused by viral transmission with COVID-19. Some of the most relevant include the following:

- Like COVID-19, gambling disorder is a disease. Not all psychological problems are considered illnesses. Only the psychological problems listed in DSM-5-TR or ICD-11 are considered mental disorders [1, 2].
- The disease is caused by the action of an external agent. The agent of COVID-19 is SARS-CoV-2, whereas the activity of betting itself is ultimately responsible for the genesis of gambling disease. This assertion is based on the guidelines in the Diagnostic and Statistical Manual of Mental Disorders (Fifth Edition Text Revision) of the American Psychiatric Association (APA), which states that “*gambling behaviors activate reward systems similar to those activated by drugs of abuse and produce some behavioral symptoms that appear comparable to those produced by substance use disorders”* (DSM-5-TR, p. 543) [1].
  On the contrary, many other mental illnesses (i.e., schizophrenia and psychotic, bipolar, obsessive-compulsive, neurocognitive, and personality disorders, etc.) are not typically caused by an external agent.
- The external agent was already in existence, but certain environmental conditions favored its propagation, which then occurred to a greater extent with a greater speed. SARS-CoV-2 jumped from other animals to humans and spread extremely quickly. In the case of gambling, it has always been present, but when economic interests and favorable regulations generate a “breeding ground,” its expansion in society is favored. Global commercial gambling has grown to be an industry of extraordinary size and power [3]. The effects of the expansion of gambling not only harm the most vulnerable people [4], but also condition government policies, affecting society in general [5].
- This turns gambling disorder from a mental health problem into a public health problem [6], since they are the environmental conditions that favor the appearance, development and spread of gambling disorder. Not all mental disorders caused by an external agent are a public health problem (i.e., trauma and stressor-related disorders, feeding and eating disorders, etc.). For that reason, gambling addiction requires policy action to prevent harm [7, 8], mainly reduce availability, make access difficult and restrict (or forbid) the commercial promotions [9].
  Second, if gambling is a disease that is transmitted due to favorable environmental conditions, which is why it has become a public health problem, it is worth asking whether the principles upon which measures to prevent the spread of COVID-19 are based would be useful in preventing gambling addiction in society.
  Prevention of COVID-19 is based on two principles: immunization against the virus and prevention of the contagion.
- Regarding immunization, for SARS-CoV-2, it is obtained through vaccination. The effect of virus inoculation in provoking the body's autoimmune response is well known in Medicine. However, there is nothing quite like it in Psychology when it comes to gambling, since gambling a bit (even "responsibly") does not prevent the onset of gambling disorder [10]. Rather, on the contrary, it favors the spread of the disease because, with responsible gambling actions, governments and gambling companies make the gambling look better [11, 12]. So, it is unlikely that immunization can be developed for any gambling addiction prevention program to immunize everyone who is exposed to the “gambling virus”. Actually, the psychological resources that could immunize anyone involved in gambling are unknown. But even if those resources were discovered, what would not be possible is to train all citizens in such skills, contrary to what has happened with the vaccination of SARS-CoV-2.
- Thus, in the case of gambling disorder, preventive strategies should rather reflect the second tier of action against COVID-19, that is, prevent gambling from affecting most people by limiting availability (supply) and accessibility (ease of access) to gambling [9]. This is especially important for the more dangerous variants of gambling, such as electronic gaming machines (EGMs) and online gambling [13, 14]. Unlike the variants of SARS-CoV-2, in the case of gambling we can identify previously where the different variants of "gambling virus" are, which would allow us to implement appropriate preventive measures for specific games. Likewise, just as there are less contagious and lethal variants of SARS-CoV-2, there are also games, such as lotteries, that are less addictive and harmful than EGMs and various types of online gambling. In the case of COVID-19, the danger posed depends on the DNA structure whereas, for gambling, the structural characteristics of the games are the most important factors [15–17]. Therefore, measures to prevent gambling addiction must be adapted to each type of game.

However, once a person has been exposed to the effects of gambling, the next phase of prevention (selective prevention) would be to control the effect that gambling has on people who risk their money; i.e., recognizing the appearance of symptoms and acting effectively in response. As with the vaccine, it is not possible to train all gamblers to carry out gambling behaviors that prevent the development of gambling disorder. It is not possible for players to develop responsible gambling behaviors if the conditions in which gambling is offered in society do not drastically change [18]. In the case of selective prevention, again it must be public health policies that must be implemented.

Probably the most effectives preventive strategies in selective prevention are to limit losses and prevent affected people’s access to gambling [18]. In these cases, governmental regulation of gambling seems essential, because those affected are not able to reduce their exposure to gambling, nor are companies interested in reducing their income, which mainly comes from people who suffer from gambling addiction [19].

Finally, once a person has been infected and suffers from gambling disorder, it is necessary to use other measures beyond access control or limit losses. Gambling disorder is a clinical phenomenon [20] characterized by a loss of control over behavior that results not only in spending excessive amounts of money but also in alterations in emotional adjustment and interpersonal relationships. Psychological treatments for gambling disorder should not only reduce or eliminate gambling behavior, but also promote other alternatives that favor a new lifestyle without gambling [21]. Behavior modification techniques have been shown to be effective in reducing or eliminating excessive behaviors, training in coping techniques and in promoting alternative adaptive behaviors [21–23]. This is the only way to immunize against the effects of the “gambling virus”, but it is not a universal prevention procedure, since it is not possible to "immunize" the entire population in this way, but only patients undergoing psychological treatment. Effective preventive measures for the entire population must be carried out through gambling policies, that is, through gambling regulation [8].

In this sense, the effect on the pattern of gambling and gambling problems of the measures carried out for the prevention of COVID-19 can guide legislators and governments on the specific measures that must be taken to prevent gambling disorder from a public health perspective [15].

In a recent systematic review of 34 studies from 12 countries [24], it was concluded an overall reduction in gambling amongst the general population during the COVID-19 pandemic at the level of the general population. However, marked increases in gambling amongst young adults (18–30 year olds) and people with pre-existing at-risk gambling. There was conflicting evidence among the different studies regarding educational, employment status or socioeconomic level.

The main objective of the research is to describe the changes in gambling patterns and addiction that have occurred in Spain one year after the lockdown was implemented to counteract the COVID-19 pandemic. The results of this study analyzed from the conceptual framework that we have just described, will serve to guide gambling policies based on public health.

The first research hypothesis is that the frequency of gambling will decrease because the measures to prevent COVID-19 also restrict access to gambling. However, such measures will not affect all types of gambling equally, only those types that take place in public spaces (e.g., gambling halls, casinos, etc.). Online gambling via electronic devices (e.g., mobile phones, computers, and tablets) will not be affected.

The second hypothesis is that the type of game is relevant when it comes to causing addiction, due to the structural characteristics of the different games. Therefore, people who play landscape gambling and online gambling (e.g., casinos, bingo, and slots online) are more likely to suffer from gambling disorder than those who play lotteries.

## Method

### Participants

In total, 2,903 people (55.6% women and 44.4% men) between the ages of 15 and 85 (Mean = 36.5; SD = 14.6) years participated in this study by responding to an Internet survey during the period from May–November, 2021. The survey was distributed over the Internet by 251 professionals and attendees of gambling addiction prevention training courses from several regions of Spain. The participants knew the objective of the research and freely agreed to participate.

### Instruments

#### Gambling participation

A survey on gambling behavior was administered. In this survey, participation in gambling before and after the measures taken to combat the COVID-19 pandemic was evaluated by self-report. The results were categorized into three groups based on the restriction conditions applied by government authorities aiming to prevent COVID-19, as follows:

- No restrictions: *online gambling*.
- Moderate restrictions: *lotteries*. There were 2 months without lottery draws at the beginning of the restriction period. After the restriction period, the lotteries returned to pre-pandemic conditions.
- Severe restrictions: *landscape gambling*. For several months access to some game types was prevented and subsequently the capacity of gaming halls was limited.

#### Gambling problems

Gambling participation and gambling problems before and after the measures taken to minimize SARS-CoV-2 virus transmission were evaluated in the same survey. To avoid response bias, two different diagnostic questionnaires were used, both of which met the necessary methodological requirements:

- Brief Problem Gambling Screen [25]. A five-item questionnaire to identify people who suffer from gambling disorder and at-risk gambling. The psychometric analysis of the scale performed with the data from this study showed adequate internal consistency (*Cronbach α* = .76).
- NORC DSM-IV Screen for Gambling Problems, NODS [26]. A 17-item yes/no scale that aims to diagnose pathological gambling according to the diagnostic criteria of the DSM-IV-TR. It was adapted to the current DSM-5 criteria. The range of the scale scores is 0–9. The psychometric analysis of the scale using the data from this study showed high internal consistency (*Cronbach α* = .94).

### Procedure

People who regularly (≥ 1–2 times per month) played different types of games based on the above categories were selected for analysis. Responses pertaining to gambling participation and the incidence of problem gambling were compared between two time points: before the implementation of preventive measures against the pandemic (March 20, 2020) and approximately 1 year later (May–November 2021), once the restrictive measures had been eliminated and it was possible to play again with relative normality.

To avoid bias in the response to the gambling addiction evaluation questionnaires, two different diagnostic questionnaires (BPGS and NODS) were used. The diagnosis of pathological gambling before the pandemic was made with the BPGS scale, while the evaluation of this disorder after the measures taken to minimize SARS-CoV-2 virus transmission was done using NODS.

## Results

### Gambling participation

Table 1 gives the percentages of people in this study who regularly played some game (> 1–2 times per month) before and after COVID-19 preventive measures were in place.

**Table 1:** Percentage of frequent players before and after the implementation of COVID-19 preventive measures

	*Before*	*After*	*% decrease*
Lotteries	1,345 (46.33%)	822 (28.32%)	38.87%
Landscape gambling	759 (26.15%)	263 (9.06%)	65.35%
Online gambling	558 (19.22%)	361 (12.44%)	35.28%

There was a reduction in frequent participation in all types of gambling, with the greatest reductions for landscape games.

A complementary way to understand the changes that occurred is to study whether current regular gamblers were also regular players before the pandemic. Table 2 shows the percentage of regular gamblers after implementation of the COVID-19 preventive measures who already were frequent gamblers, considering the different game types.

**Table 2:** Percentage of frequent players after the implementation of COVID-19 preventive measures who were already frequent players

	Lotteries	Landscape gambling	Online gambling
Were frequent players before	94.77% (779/881)	88.59% (233/263)	88.37% (319/361)

The type of gambling with a lower percentage of new gamblers was lotteries (5.33%). No differences were found in the percentage of new gamblers between landscape and online gambling.

### Gambling problems

#### Differences according to sex

The percentage of women and men affected by gambling problems (risk gambling and gambling disorder) in this study are indicated in Table 3.

**Table 3:** Percentage of women and men affected by gambling problems^a^

	*Risk gambling*	*Gambling Disorder*
Women	10.9%	5.9%
Men	20.4%	10.5%
*Total*	*15.0%*	*8.0%*

^a^Measured by NODS [26]

Women who participated in this study reported fewer gambling problems than men, both in terms of gambling disorder (*χ*^*2*^ = 20.65; *p* < 0.001; *φ* = 0.09) and risk gambling (*χ*^*2*^ = 45.77; *p* < 0.001; *φ* = 0.13).

#### Changes in gambling disorder incidence

Regarding gambling disorder, Table 4 lists the percentages of participants who exhibited gambling disorder before and after the implementation of pandemic-related preventive measures.

**Table 4:** Percentage of participants with gambling disorder (GD) before and after the implementation of preventive measures

	Without GD after n (%)	GD after n (%)	Total (n)
Without GD before^a^	2,655 (93.6%/99.4%)	181 (6.4%/78.4%)	2,836
GD before^b^ *Total*	17 (25.4%/0.6%) *2,672*	50 (74.6%/21.6%) *231*	67 *2,903*

^a^Measured by BPGS [25]

^b^Measured by NODS [26]

More survey participants exhibited pathological gambling after the pandemic than before the restrictive measures were taken (231 vs. 67). Most people who exhibited gambling disorder before the pandemic also manifested it later (74.6%), whereas only 6.4% of those who did not engage in pathological gambling before the pandemic developed gambling disorder after the measures were implemented. Of the people with gambling disorder after the pandemic, 21.6% had a gambling disorder before, while only 0.6% of those without current gambling disorder showed pathological gambling before the restrictive measures were taken. The difference in these percentages was significant (*χ*^*2*^ = 416.21; *p* < 0.001; *φ* = 0.38).

#### Gambling disorder with regard to the different types of gambling

Regarding gambling disorder among those who frequently engaged in different types of gambling, we summarize the main results in Table 5. Our results also consider whether gamblers regularly partake in a single type of gambling (lotteries, landscape, or online gambling) or several types.

**Table 5:** Gambling problems^a^ (at-risk gambling and pathological gambling) according to the type of gambling

	Without gambling problems	Gambling disorder	Gambling disorder (single type of gambling)
Lotteries	527 of 822 (64.1%)	125 of 822 (15.2%)	19 of 591 (3.21%)
Landscape gambling	55 of 263 (20.9%)	124 of 263 (47.1%)	7 of 40 (17.50%)
Online gambling	77 of 361 (21.3%)	150 of 361 (41.6%)	21 of 115 (18.26%)

^a^Measured by NODS [26]

## Conclusions and discussion

The objective of the research was to analyze the effect on gambling behavior and gambling disorder that the measures to restrict access to public places that were taken to avoid COVID. Some preventive strategies based on the the conceptual framework and the results of this research are suggested.

The results were partially consistent with the hypotheses, because the main reduction in gambling frequency occurred in landscape gambling, which is the type of gambling that suffered the most from restrictive access measures. There was also a reduction in the frequency of lottery gambling, although the measures were temporary. These results are congruent with other research showing a reduction in gambling frequency during lockdown measures [27–30]. Unexpectedly, there was also a decrease in the frequency of online gambling, even though it was widely promoted and advertised and there was a very noticeable increase in spending on online gambling during this period of time [31]. This result may be due to the fact that this research is not an epidemiological study, in which it is intended to evaluate the prevalence of gambling behavior before and after the measures adopted to prevent COVID-19, but the changes produced in the gambling behavior after the implementation of such measures. For that reason, only the results for people who gambled regularly were analyzed.

The percentage of people who participated in different betting games regularly decreased markedly after preventive measures were taken, especially in games that take place in gambling venues or public places with slot machines, as is the case for bars and restaurants in Spain. Therefore, it seems that the measures taken globally to prevent the spread of SARS-CoV-2 could have had an effect in reducing the frequency of gambling, because at one year after implementation of the most restrictive measures, the percentage of people who frequently participate in gambling seemed to be lower. This is a positive outcome in terms of preventing gambling addiction, because frequent gambling is one of the main factors favoring the development of gambling disorder.

This reduction occurred especially in games that take place in venues (gambling halls, bars, etc.) where gamblers have to be physically present and can spend several hours playing at a time. Many of the people who gambled frequently stopped doing so, especially those who previously went to gambling venues or gambled in public places. It is likely that the increase in new frequent players will be at a rate similar to that found in this study, in the range of 1.5–3%. With the restrictive measures taken against the expansion of COVID-19, many of the frequent gamblers who had not yet consolidated the habit of gambling or developed gambling disorder before the pandemic may not return to a frequent pattern of gambling when conditions return to normal, at least for now. If this has helped people to modify their lifestyles, it would have served as a positive preventive measure against gambling addiction.

When it comes to gambling disorder, the majority of those who currently suffer from pathological gambling had already suffered from it before the implementation of COVID-19 measures, whereas only a small percentage of people who did not currently suffer from gambling disorder exhibited symptoms before the pandemic. This may be related to the restrictive measures implemented to prevent the spread of SARS-CoV-2 being useful in also preventing the promotion of new cases of pathological gambling. However, the measures were not sufficient to solve the problem for those who were already suffering from gambling disorder. That is to say: pathological gamblers need specialized treatment. These results are consistent with other investigations that have found no significant reduction in gambling frequency for those who were most engaged in gambling pre-lockdown, especially pathological gamblers [32].

Not all types of gambling were equally affected by the restrictive measures. Hence, when analyzing the changes associated with pathological gambling after the implementation of preventive measures, differences in the addictive potential of the different types of gambling (landscape, lotteries, and online gambling) must be considered. The addictive potential of the different types of gambling is evidenced when comparing the percentage of pathological gamblers in the groups that regularly gamble in only one type of game. Regular lottery players had five to six times lower rates of gambling disorder and risky gambling behavior compared to those who frequently played landscape gambling or online gambling. Approximately 80% of the people who regularly played these games were found to suffer from pathological or at-risk gambling, which is a very high figure in our opinion. This is due to the structural characteristics of electronic games [15–17] (landscape gambling) and online gambling [33].

For this reason, we consider it necessary to take measures restricting access to these specific games (EGMs and online gambling) to prevent the development of pathological gambling in society, i.e., to avoid the spread of the “gambling virus.” However, once a person has been infected and suffers from gambling disorder, it is probably necessary to use other therapeutic measures beyond access control itself.

This study had some limitations. Like most studies that have analyzed the effect of COVID-19 on gambling behavior [24], it is a cross-sectional study, rather than longitudinal, and may have been affected by recall bias. Another limitation is that we focused on self-report data. Although this study was carried out with general population (that is, it was not a study with clinical population), the sample is not random and it was selected by addiction prevention professionals. Therefore, it is not an epidemiological study and, accordingly, data on the prevalence of gambling disorder in general population cannot be concluded. The fact that there were more participants in the survey with problem gambling after the pandemic than before does not necessarily mean that there was an increase in the incidence of pathological gambling, but rather that people with a current problem with pathological gambling were more interested in responding to the survey. However, most of the analyses conducted on problem gambling have been conducted, not with the general population, but with people who frequently participate in gambling. This allows us to assume that the conclusions deduced here about differences in the risk of addiction for the different types of gambling and the differential impacts of preventive measures are somewhat valid. However, the conclusions must be treated with caution because it is a correlational study and, although the number of respondents is high, it lacks an experimental design.

The main conclusions of this study are the following:

- Conceiving of gambling as a virus has important implications for the prevention of gambling disorder. Although it is not possible to implement universal vaccination for consequences of gambling, such that people are immune to it, some measures taken to prevent the spread of SARS-CoV-2 based on lockdown and social distancing may be also useful to prevent gambling disorder. Some examples could be regulating long distances between bookies and schools or among gambling rooms, authorizing EGMs only in gambling rooms and casinos (not in bars or restaurants), etc [18].
- Just as there are less contagious and lethal variants of SARS-CoV-2, there are also gambling games, such as lotteries, that are less addictive and harmful than EGMs and various types of online gambling. In the case of COVID-19, the danger posed depends on the DNA structure, whereas for gambling the structural characteristics of the games are the most important factors. Some preventive measures could include the modification, by law, of some parameters of the games to make the game virus less addictive. For example: restrictions on gambling speed; delaying the time between the bet and the outcome; reduction of maximum bet size; diminishing the percentage of win; posting the payoff probabilities; reducing the frequency of “near-miss” outcomes on EGMs; or prevent, through the use of gambling smart cards, gamblers from losing large amounts of money [18].
- As in the case of virus infections, measures to prevent the spread of disease must also be adapted to social and environmental conditions, placing special emphasis on the most socially and economically vulnerable groups. Therefore, gambling advertising and commercial promotions must be limited. Even in capitalist societies, public health must take precedence over the economic benefits of companies. Paraphrasing the philosopher Michel Sandel, moral limits must be applied to the market [34]; in the case of gambling virus, such moral limits should enforce to gambling companies.

## Data Availability

For security reasons, the datasets generated and/or analysed during the current study are not publicly available due none Gambling and Technological Addictions Research Unit databases can be found on the Internet (Management agreement), but are available from the corresponding author on reasonable request.
